# Hysteria in Childhood

**Published:** 1879-11

**Authors:** 


### SELECTIONS.

### HYSTERIA IN CHILDHOOD.

In the *Pacific Medical and Surgical Journal* (September, 1879) Dr. Buckley mentions, among other cases of hysteria, one of a child which in all particulars is similar to those referred to under our Translations. A child of ten years lies in bed with the right leg drawn up; says she has pain in knee and hip-joints; seems a healthy, well-developed child, and had been very fond of play until ten days ago (March 20, 1879), when she complained of the leg, and they believed she had in some way hurt herself, and suspected hip disease. I found the temperature of knee and hip-joints of the affected leg the same as the other. In striving to move the leg a little, the hip-joint showed no motion; on the contrary, the spinal column moves with the motion of the leg. The flexion of the leg, however, was not that generally accompanying hip-disease, for the heel was drawn up almost to the nates.

The child spoke little, looked quite grave, and seemed to suffer little or no constitutional distress. I informed the parents that there was no disease of the hip-joint, but a serious trouble of the spine, believing the case to be one of progressive infantile paralysis, more especially as the child had had a severe attack of diphtheria within a year. Next day I learned that the child had also lost her speech, and this, while it might seem to confirm my original opinion, had the very opposite effect, for the reason that there was no paralytic affection of the left leg, none of the right arm, none of the muscles of deglutition, the appetite remaining unaltered. Ordered the spine rubbed with a mixture of chloroform and camphor liniment.

The following afternoon I found that she had recovered the power of speech, but the leg was nowise altered.

Then with a view of testing a strong suspicion, I pulled down the bed-clothes, and in a very positive tone told the child to "straighten out that leg." She looked at me quite vacantly, but took no heed of my injunction. After this I opened a pocket bistoury, and told her if she did not straighten her leg I should have to cut off part of it. Still not the slightest effort at moving, until with the fine point of the bistoury I gave her one or two pricks in quick succession, when she jumped out of bed, and ran around to her brother for protection, very much to the stupefaction of both parents, who witnessed the performance. After this no more lameness, and the well-being continues to the time of this writing.

